# Active and resolved HCV infections among people with new HIV-1 diagnoses in Germany, 2009–2019

**DOI:** 10.1186/s12879-025-12025-8

**Published:** 2025-11-05

**Authors:** Daniel Ivanusic, Amare Eshetu, Hussein Al-Shehabi, Andrea Hauser, Patrycja Klink, Daniel Schmidt, Uwe Koppe, Barbara Gunsenheimer-Bartmeyer, Viviane Bremer, Norbert Bannert

**Affiliations:** 1 Unit 18 HIV and Other Retroviruses/Sexually Transmitted Bacterial Pathogens (STI) and HIV, Department of Infectious Diseases, Robert Koch Institute, Nordufer 20, 13353 Berlin, Germany; 2 Unit 34 HIV/AIDS, STI and Blood-Borne Infections, Department of Infectious Disease Epidemiology, Robert Koch Institute, Berlin, Germany

**Keywords:** Hepatitis C Virus (HCV), Human Immunodeficiency Virus (HIV), People Who Inject Drugs (PWID), Men who have Sex with Men (MSM)

## Abstract

**Background:**

HIV and HCV share similar routes of infection. Individuals carrying both viruses experience a faster progression of the liver disease. We have analyzed people with new HIV-1 diagnoses in Germany for active or resolved HCV infection for over ten years. The time period covers the introduction of direct acting antivirals (DAA), a paradigmatic shift in HCV therapy.

**Methods:**

A central component of the HIV surveillance in Germany is the notification of new diagnoses. Residual blood samples from 16,539 people with new HIV-1 diagnoses reported between 2009 and 2019 were examined for HCV antigen and/or antibodies. Reactive cases were further investigated for active or resolved HCV infection by RT-qPCR. The results were analyzed with socio-demographic information from the notification forms.

**Results:**

The study includes samples from 48.0% of all notified HIV-1 diagnoses. The seroprevalence of cases with HCV antigen, antibodies or both, representing active and resolved infections was 6.2% with stable seroprevalence. The average proportion of resolved infections among those was 33.0% with a significant increase since the introduction of DAAs in 2012 (p_Trend_2012-2019_ = 0.028) reaching 48.2% in 2019. The highest proportion of active and resolved cases (73.7%) was found in people who inject drugs (PWIDs). This transmission group had the lowest percentage of resolved infections with 29.4%. The proportion of active and resolved cases in persons with heterosexual mode of transmission (HET) and in men who have sex with men (MSM) was 3.8% and 2.6%, respectively. The peak percentage of resolved infections was found in MSM (40.0%), followed by HET (36.6%). The proportion of active and resolved cases among individuals with non-German origin was higher than in people with German origin (8.8%, versus 4.3%; *p* < 0.001) and the proportion of resolved HCV infections lower (27.8% versus 34.0%; *p* = 0.027).

**Conclusions:**

The proportion of resolved HCV infections among people newly diagnosed with HIV-1 increased after the introduction of DAAs in Germany. The high prevalence and the low proportion of resolved HCV infections reveal that unmet diagnostic and therapeutic needs exist among PWIDs. The higher proportion of active and resolved cases among individuals of non-German origin particularly requires greater public health attention.

**Supplementary Information:**

The online version contains supplementary material available at 10.1186/s12879-025-12025-8.

## Introduction

WHO estimates that in 2022 about 50 million individuals worldwide were living with the hepatitis C virus (HCV) and 39 million with the human immunodeficiency virus (HIV) [1, 2]. These pathogens have overlapping modes of transmission and affected populations. Reported coinfection rates differ widely depending on the geographical region, the studied population and the sampling period [3]. According to the most recent meta-analysis at global level that includes 783 studies conducted in 2002–2015 about 2.3 million individuals were coinfected of whom 60% are people who inject drugs (PWIDs) [4]. Transmission by sharing of needles and other paraphernalia with contaminated blood is effective for both viruses especially for HCV which is about 10-times more efficiently spread by this means than HIV [5]. In contrast, transmissibility by sexual activity and rates of vertical transmission are much lower for HCV. On the other hand, HIV infection increases the probability of HCV acquisition through both of these routes [6, 7]. The odds of an HCV infection are 6-times higher in people living with HIV (PLWH) compared to the HIV-negative population [8]. Besides PWIDs, men who have sex with men (MSM) are a population at higher risk and outbreaks of HCV infections among MSM living with HIV are well documented [9, 10].

The synergistic effect of both viruses has ramifications on the morbidity caused by each of the pathogens but the impact of the lentivirus on the HCV-related disease is much more severe. HIV coinfection increases the already high HCV chronification rate of 70–80% to up to 95%, depending on the CD4^+^ T-cell count [11]. It also accelerates progression to liver fibrosis, end stage liver disease or hepatocellular carcinoma and death [12]. The effects are mediated by direct mechanisms affecting hepatocytes, hepatic stellate cells and Kupffer cells as well as by indirect mechanisms resulting from increased microbial translocation in the gut and immune activation in PLWH (reviewed in [8]). As a consequence, functional liver cells are lost by apoptosis and fibrogenic processes are triggered. Vice versa, HCV coinfection is a major driver of non-AIDS-related morbidity in PLWH [13]. HCV-caused chronic liver inflammation augments expression of various exhaustion markers on CD8^+^ T-cells resulting in less potent activity against HIV infected cells [14]. Moreover, the size of the HIV reservoir in resting CD4^+^ T cells seems larger in individuals with persisting HCV infection compared to individuals with HIV monoinfection [15]. Regarding HIV and HCV treatment aspects of coinfected patients, the introduction of the first generation of direct-acting antivirals (DAAs) in 2012 and even more, the introduction of the second generation of these drugs two years later, were major improvements. In the interferon (IFN)-era treatment and cure in dually infected subjects was challenging since IFN-based regimens had multiple side-effects in these patients and a lower rate of sustained viral response was observed [16]. The availability of less liver toxic HIV drugs reduces risks as well. Since second generation DAAs are available, the predictors of failure to achieve HCV elimination by DAAs and improvement of liver fibrosis markers in dually infected patients are not different from those in monoinfected [17, 18].

Despite a general availability of effective therapies for both chronic infections, mono- and coinfections remain a significant public health concern. The German government has committed to the WHO agenda for elimination of sexually transmitted and blood-borne infections as a public health threat by 2030 [19] with a national strategy including HCV and HIV [20]. In order to add to the knowledge of epidemiologic trends and other aspects of HIV/HCV coinfection in the country we have analyzed a large proportion of notified new HIV diagnoses for HCV coinfection reported between 2009 and 2019. The focus is on groups at risk and the period encompasses the introduction of DAAs and phases with increased migration into the country.

## Methods

### Sample collection

The Robert Koch Institute (RKI) receives notification of new HIV-1 diagnoses for surveillance purposes from diagnostic laboratories and physicians as specified in the German law (Infection Protection Act). In this context, a network of approximately 80 diagnostic laboratories across Germany reporting a significant proportion of the cases is submitting residual serum or plasma along with the notification form for supplemental monitoring, as described earlier [21–23]. Data on age, gender, mode of transmission and the region of origin were obtained from the associated HIV notification form for our study. Between January 1^st^ 2009 and December 31^st^ 2019 all samples were submitted as dried serum or plasma spots (DSS/DPS) on filter paper (Whatman 903 GE Healthcare Bio-Science Corp, Westborough, USA). All tests were performed on the same platforms and under the same protocols at the RKI.

### Elution of antibodies from DSS/DPS

One filter spot containing 100 µL of dried serum was incubated overnight in 400 µL of elution buffer (0.05% Tween-20 (Carl Roth), and 3% fetal calf serum (FCS, Gibco)), dissolved in 1x PBS at 4 °C. The filter was subsequently removed and the extracted material stored at 4 °C for later processing. Extraction results in a 1:5 dilution of the serum or plasma [23, 24].

### Enzyme-linked immunosorbent assay (ELISA)

For the identification of cases with resolved or active HCV infection we have previously evaluated two potential ELISA systems for detection of HCV antigen and antibodies among HIV/HCV infected patients in dried serum/plasma spots [24]. As a consequence of this validation we have chosen the commercially available HCV specific IgG ELISA (Monolisa Ag-Ab Ultra V2, BioRad) and used it according to the manufacturer’s instructions analyzing 50 µL of the filter-extracted material.

### Western blot

For a qualitative detection of HCV antibodies in DSS/DPS eluates the recomLine HCV IgG assay (Mikrogen Diagnostik, Neuried, Germany) was used. 20 µL of eluates were analyzed according to the instructions of the manufacturer.

### Extraction of total RNA

To enable discrimination of active HCV infections (HCV RNA-positive) from resolved HCV infections (HCV RNA-negative), RNA extraction was carried out using magnetic beads (NucliSENS easyMAG, Biomerieux) in a semi-automated process from ELISA-reactive samples. In this preparation, two filter spot samples were incubated in 1.8 mL lysis buffer for one hour at room temperature on a shaker. HCV RNA extraction followed the manufacturer’s instructions, resulting in an elution volume of 60 µL. The gained RNA was subsequently aliquoted and stored at −80 °C.

### cDNA synthesis and quantitative real-time pcr (RT-qPCR)

Detection and quantification of HCV RNA was performed using an in-house quantitative real-time PCR (RT-qPCR) assay targeting the conserved 5′ untranslated region (5′ UTR) of the HCV genome. The reaction was carried out using the SuperScript® III Platinum® One-Step qRT-PCR Kit (Invitrogen) in a total volume of 12 μL per reaction. The master mix consisted of 6 μL of 2x reaction mix, 0.5 μL of forward primer HCV-255 (10 μM), 0.5 μL of reverse primer HCV-256 (10 μM), 0.2 μL of probe HCV-TM5 (10 μM), 0.2 μL of enzyme mix, and 0.6 μL of nuclease-free water. To each reaction 4 μL of RNA template was added. The following primers and probe were used: HCV-255_f: 5´-AGYGTTGGGTYGCGAAAG-3´, HCV-256_r: 5´-CACTCGCAAGCRCCCT-3´, Probe HCV-TM-5: FAM-5′-CCTTGTGGTACTGCCTGA-3′-MGB [25]. Each sample was tested in duplicate. For quantification a standard curve was generated in every run using plasma samples containing HCV genotype 1a at concentrations ranging from 10^1^ to 10^6^ international units (IU) per mL. HCV-negative plasma served as a negative control. The viral RNA concentration in each sample was determined by extrapolation from the standard curve. Thermal cycling was performed on a LightCycler 480 II (Roche) using the following program: an initial cDNA synthesis step at 50 °C for 15 minutes, followed by an initial denaturation at 95 °C for 2 minutes. This was followed by 45 amplification cycles, each consisting of denaturation at 95 °C for 15 seconds and annealing/elongation at 58 °C for 45 seconds (with a ramp rate of 1°C/s). Samples with Ct-values below the negative control and in the range of the standards were regarded as positive.

### Statistical analysis

Statistical analyses including trend analyses were performed using Microsoft Excel (Microsoft Inc.). For continuous variables means and 95% confidence intervals (95% CI) were computed. Chi-square tests were used to analyze differences in population proportions between two groups. P-values for linear regression parameters were calculated by using the data analysis add-in for Excel. For comparison of the frequency of a subpopulation in the study population and the frequency in the notifications a weight has been calculated by dividing the proportion of the subpopulation among all notifications with the proportion among the DSS/DPS cases [23].

## Results

### Analyzed samples and characteristics of the study population

**Fig. 1:**
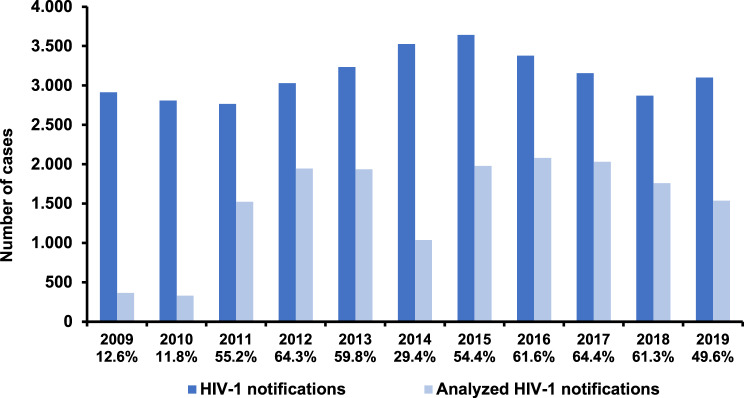
Yearly coverage of samples from HIV-1 notifications that were analyzed in the study period. The analyzed percentage of notified cases is given below the year

In the eleven years between 2009 and 2019 the RKI has received 34,436 notifications of validated new HIV-1 diagnoses in Germany [26]. DSS/DPS prepared from residual blood were available from 16,539 of these cases (48.0%). The coverage varied and was the lowest with 11.8% in 2010 and the highest with 64.4% in 2017 (Fig. 1). The study population (analyzed notifications) consisted mostly of males (80.3%, 13,285/16,539) (Table 1). The most frequently reported mode of transmission was MSM (55.3%, 9,153/16,539) followed by persons with heterosexual mode of transmission (HET; 23.8%, 3,931/16,539). A total of 605/16,539 (3.7%) were samples from PWIDs. Information concerning the country of origin was only available for notifications between 2011 and 2019. Of the 15,841 analyzed DSS/DPS from new HIV-1 diagnoses in this period of time, 57.6% (9,128/15,841) of the individuals originated from Germany, 34.7% (5,503/15,841) were non-Germans and in 7.6% (1,210/15,841) the information on the country of origin was not reported. To assure comparability of the share of notifications with available DSS/DPS with all notifications we calculated the weights. For all subgroups with reported data on gender, transmission mode and country of origin the weights ranged from 0.94 to 1.02 which is consistent with less than 10% discrepancy (Table 1). Therefore, no weight adjustments were necessary for subsequent analyses.

**Table 1:** Characteristics of notified cases and the study population

Category	Overall Notifications	Notifications with DSS/DPS samples	Weighting
	N (%)	N (%)	
Male	27,910 (81.0)	13,285 (80.3)	1.01
Female	6,469 (18.8)	3,235 (19.6)	0.96
Diverse/Not reported^a^	57 (0.2)	19 (0.1)	1.44
MSM^b^	18,584 (54.0)	9,153 (55.3)	0.98
HET^c^	7,702 (22.4)	3,931 (23.8)	0.94
PWID^d^	1,293 (3.7)	605 (3.7)	1.02
Other/Not reported^a^	6,857 (19.9)	2,850 (17.2)	1.55
**Total (2009–2019)**	**34,436**	**16,539 (48.0**^**e**^)	
Germany	16,825 (58.6)	9,128 (57.6)	1.02
Abroad	9,590 (33.4)	5,503 (34.7)	0.96
Not reported	2,296 (8.0)	1,210 (7.6)	1.04
**Total (2011–2019)**^**f**^	**28,711**	**15,841 (55.2**^**e**^)	

^a^Due to low numbers these groups were merged for reasons of data protection

^b^Men who have sex with men

^c^Persons with heterosexual mode of transmission

^d^People who inject drugs

^e^Proportion of all analyzed notifications in the indicated period of time

^f^Before 2011 country of origin was not reported

### Prevalence of active and resolved HCV coinfections

**Fig. 2:**
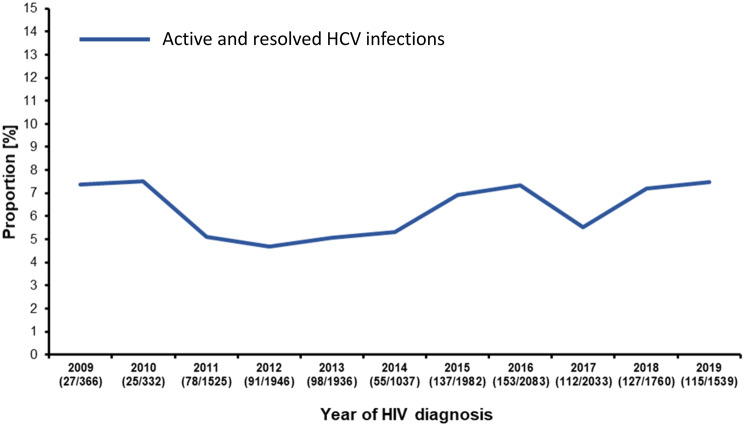
Prevalence of cases with active and resolved HCV infections. Proportion of cases positive for HCV antigen, antibody or both per year over the study period. The number of positive cases and the total number of analyzed samples is given below the year

Using a previously validated ELISA system [24], we screened the received 16,539 samples of new HIV-1 diagnoses for HCV antigen (HCV capsid protein) or HCV antibodies. 1,062 samples were ELISA-reactive. In order to identify falsely-reactive samples, we isolated RNA from 961 samples (90.5%) of which material was available (Additional file 1). Of those, 614 were positive for HCV RNA and 347 were negative for HCV-RNA. These HCV RNA-negative samples were subsequently tested by an HCV-specific Western blot. 44 of the HCV-RNA negative samples were also negative in the Western blot. These cases were therefore regarded as falsely-ELISA-reactive [27] (Additional file 1) leading to 1,018 cases with HCV antigen, antibody or both, representing all of the active and resolved HCV coinfections among the 16,539 analyzed samples (6.2%, 95% CI 5.4%-7.0%). The annual proportion varied slightly from 7.5% in 2010 up to 4.7% in 2012 without showing a trend (*p* = 0.562) (Fig. 2).

**Fig. 3:**
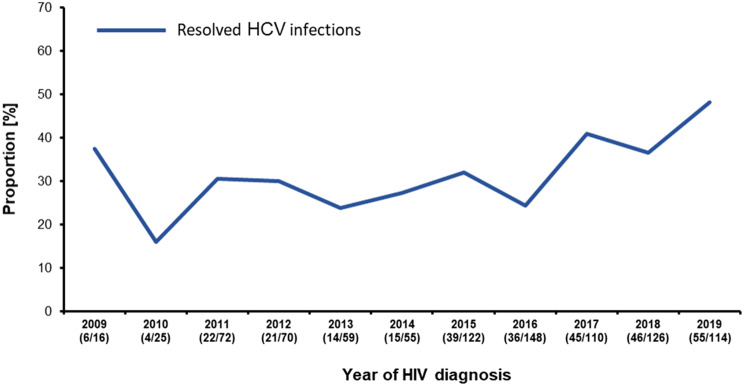
Proportion of resolved infections among cases positive for HCV antigen, antibody or both. The annual analyses were performed with 917 samples in total for which RNA for PCR testing was available. The proportion of HCV-RNA-negative cases is given below the year

Moreover, we aimed to determine the proportion of samples with resolved infection by analyzing the aforementioned 1,018 cases for HCV-RNA. Material for RNA-isolation was available from 917 of the cases. HCV-RNA has been identified by PCR in 67.0% (614/917). Thus, 33.0% (95% CI 26.8–39.2) of the people with new HIV-1 diagnoses in 2009–2019 were able to resolve a previous HCV infection. Furthermore, since first generation DAAs were introduced in 2012 we analyzed the years 2012–2019 in addition. From 2012 on, the proportion of resolved infections among people with new HIV diagnoses did increase (p_Trend_2012-2019_ = 0.028) and since 2016 the dynamic of the increase gained momentum (Fig. 3).

### HCV coinfection in males and females

Next, we set out to determine differences in HCV coinfections with respect to the gender. From almost all (99.9%) of the analyzed cases information on the gender was available. Most of the samples came from males (13,285/16,539, 80.3%) and 3,235/16,539 (19.6%) were from females (Table 1). A similar proportion of men (798/13,285, 6.0%) and women (219/3,235, 6.8%) were active or resolved HCV cases (*p* = 0.105). There was also no significant difference in the proportion of resolved HCV coinfections among the two main genders. While 233/718 (32.5%) ELISA-reactive men were HCV-RNA negative, 70/198 (35.4%) of the reactive samples from women were resolved infections (*p* = 0.441).

### HCV coinfection stratified by HIV-1 transmission group

**Fig. 4:**
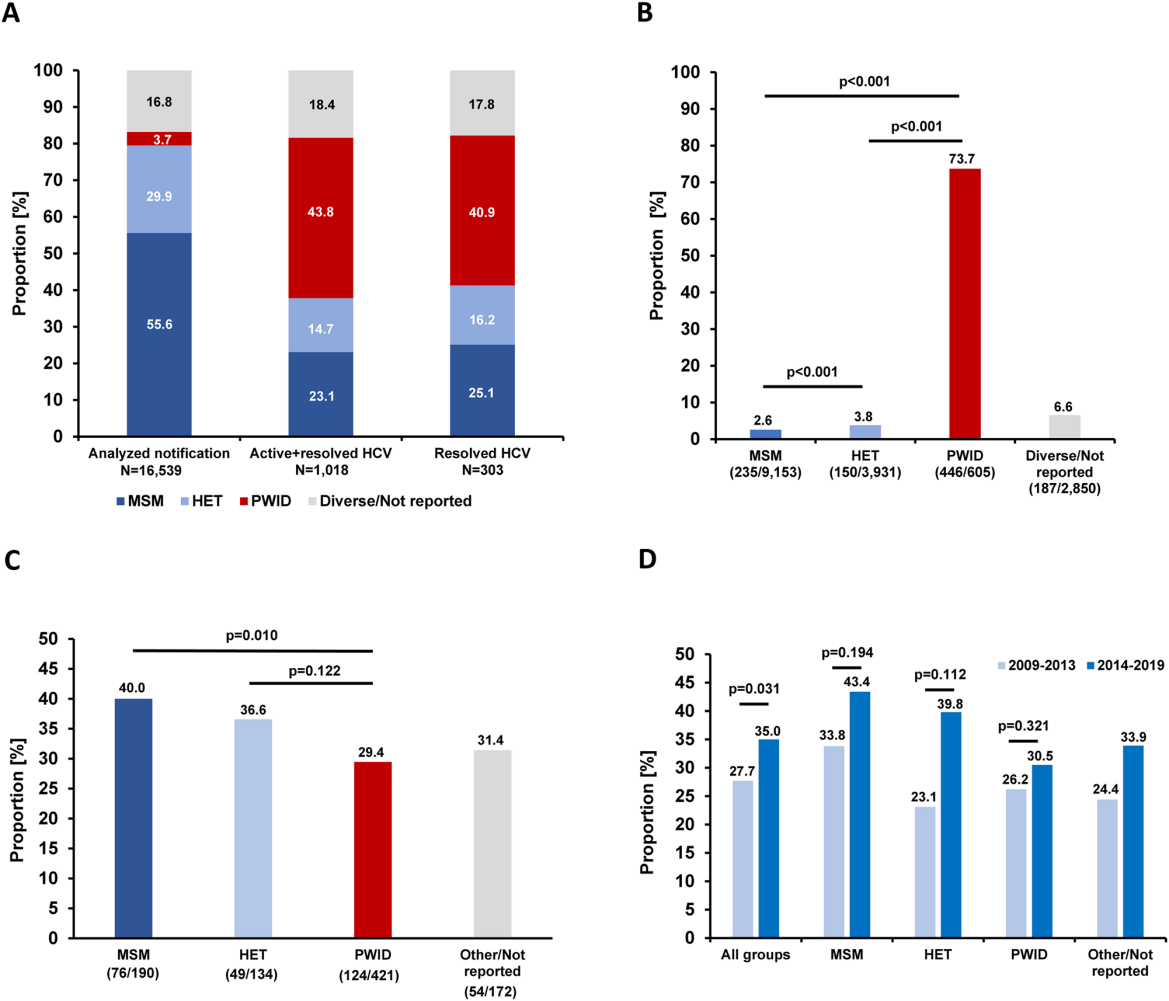
Analysis of HCV coinfections stratified by transmission groups. (**A**) Percentage of major transmission groups among all analyzed samples, among active and resolved HCV cases and among resolved HCV infections. (**B**) Proportion of active and resolved HCV cases within each transmission group. (**C**) Proportion of resolved infections within all HCV-positive cases of each transmission group. (**D**) Proportion of resolved infections among all HCV-positive cases before and after introduction of second generation DAAs in 2014

To explore and compare the risk for HCV coinfection associated with key HIV-1 transmission groups, the self-reported information on the presumed mode of HIV-1 transmission was utilized. In total 82.8% of the analyzed samples came from MSM, HET and PWIDs. The highest differences were found for the group of PWIDs (Fig. 4A) While only 3.7% (605/16,539) of the analyzed samples were from PWID, 43.8% (446/1,018) of the active and resolved HCV positive cases were observed among this group. Samples from PWID are also most frequent among the cases with resolved HCV infection (40.9%, 124/303).

We then studied the proportion of cases with ongoing or previous HCV infection and the proportion of resolved HCV infections in each of the transmission groups. The by far highest percentage of active and resolved HCV cases has been detected in PWIDs with 73.7% (446/605) and the lowest in MSM (2.6%, 235/9,153) (Fig. 4B). PWIDs had the lowest proportion of resolved infections in the study period (29.4%, 124/421) and MSM the highest (40.0%, 76/190) (Fig. 4C). In order to elucidate a presumed effect of the very potent second generation DAAs since their approval in 2014 we compared the proportion of resolved infections among the HIV-1 new diagnoses in the period before and after their release. Although the cumulative proportion of resolved infections in the later study period (2014–2019) was significantly higher (*p* = 0.031), the difference in each of the transmission groups did not reach statistical significance (*p* > 0.05) (Fig. 4D).

### Prevalence of HCV among HIV-1 new diagnoses stratified by origin

**Fig. 5:**
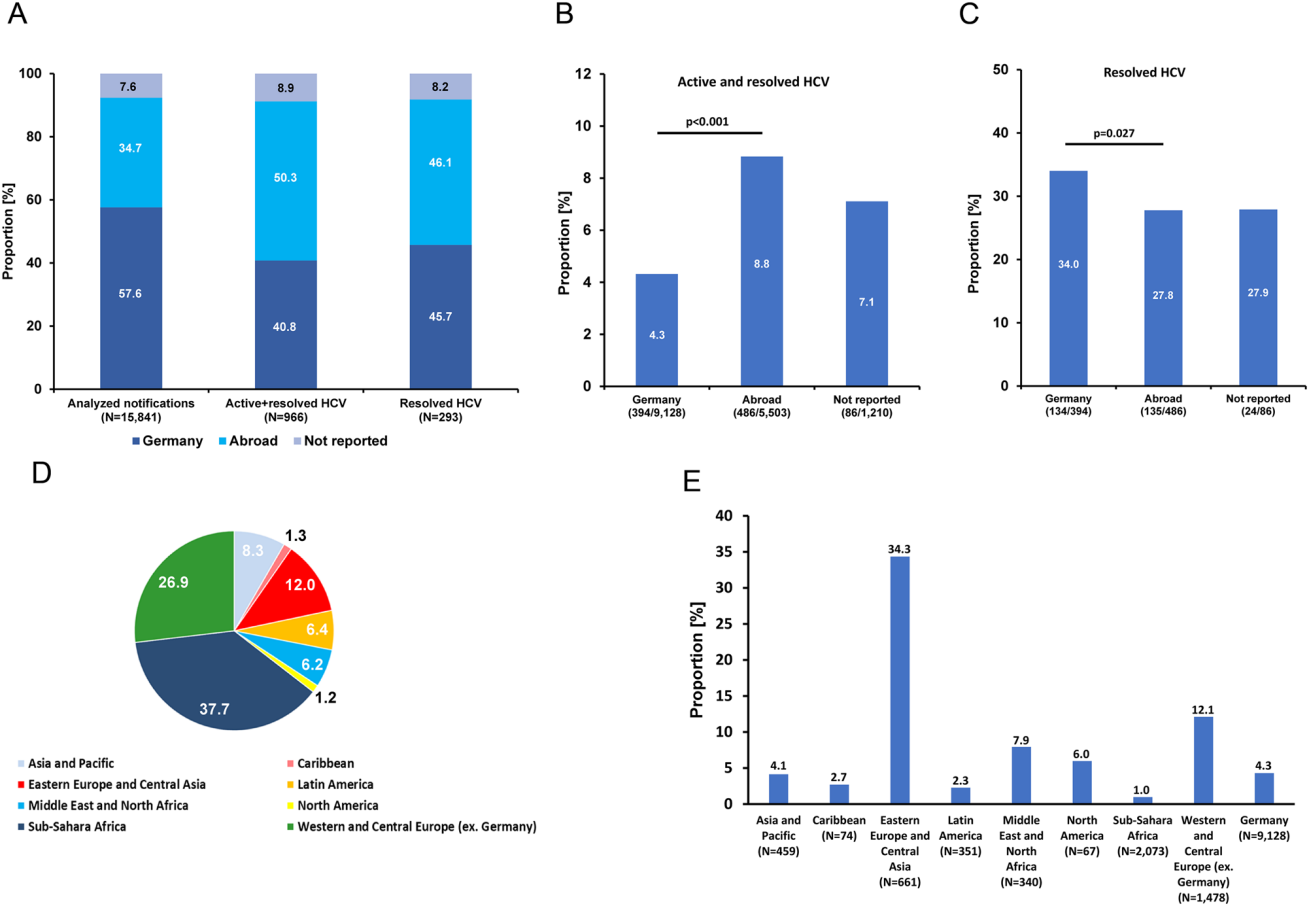
Analysis of HCV coinfections stratified by German and non-German (abroad) origin. (**A**) Proportion of cases with German and non-German origin within the analyzed notifications, all HCV-positive cases and resolved HCV coinfections. (**B**) Percentage of active and resolved HCV cases within cases of German and non-German origin. (**C**) Percentage of resolved HCV coinfections among Germans and non-Germans. (**D**) Regional origin of the non-German cases. The numbers are the percentage among all cases with non-German origin (N=5,503). (**E**) Proportion of active and resolved cases by regional origin

We subsequently explored the samples from 2011 to 2019 using information on the country of origin. In 2009 and 2010 this information has not been reported. The results obtained show that 34.7% (5,503/15,841) of the analyzed reports were from individuals with non-German origin but their share among all active and resolved HCV cases was 50.3% (486/966) (Fig. 5A). This higher share reveals a higher frequency of active or resolved HCV coinfections in individuals of non-German origin with new HIV-1 diagnoses compared to individuals with German origin. Furthermore, the proportion of cases with German and non-German origin was similar among the 293 resolved HCV-infections in the analyzed period of the study (45.7% German versus. 46.1% non-German origin).

In agreement with these findings, a further analysis with stratification by country of origin show that the prevalence of active and resolved HCV infections in individuals of non-German origin was about twice as high as in people with German origin (8.8%, 486/5,503 versus 4.3%, 394/9,128; *p* < 0.001) (Fig. 5B). Resolved HCV coinfections on the other hand were significantly less frequent in the group of non-German origin (27.8%, 135/486) compared to individuals with German origin (34.0%, 134/394) (*p* = 0.027) (Fig. 5C).

A closer look into the regional origin of the 5,503 cases of individuals from abroad revealed that the majority were from Sub-Sahara Africa (37.7%) followed by Western/Central Europe (excluding Germany) (26.9%) and Eastern Europe/Central Asia (12.0%) (Fig. 5D). Analyzing the active and resolved HCV samples, we found the highest proportion of reactive cases in the group of individuals originating from Eastern Europe/Central Asia (34.3%) followed by Western/Central Europe (excluding Germany) with 12.1% (Fig. 5E). In the largest group (Sub-Sahara Africa origin), the proportion of active and resolved HCV cases was only 1.0% (Fig. 5E). The 661 cases in the Eastern Europe/Central Asia group were mainly from countries of the former Soviet Union including Russia (301 cases) and the Ukraine (147 cases) with a very high prevalence of active and resolved HCV cases of 30.9% and 32.0%, respectively. These samples contain high proportions of cases from PWIDs. Of the 301 cases with Russian origin, 58 (19.3%) were from PWIDs and of the 147 cases with origin in the Ukraine 22 cases (15.0%) were from PWIDs while the proportion of PWIDs in the entire study was 3.7% (Table 1).

## Discussion

A critical step towards HIV and HCV incidence reduction and elimination goals is having a clear understanding of past and ongoing epidemiological developments in severely affected subpopulations. This is essential for accurate estimations of national disease burdens and for the development of well-targeted and effective public health strategies, addressing operative health-service needs.

Due to largely similar transmission routes HIV/HCV coinfections are common and frequent in subpopulations including PWIDs, MSM and people from regions with higher HIV and HCV prevalence [4, 28, 29]. Germany is considered a low prevalence country for both of these viruses causing sexually transmitted diseases with reported seroprevalence rates in the general population of about 0.1% for HIV and 0.2%-1.9% for HCV [28, 30–32]. Data on HIV/HCV coinfections in the general German population or in subpopulations are scarce [29]. In order to gain insight on HIV/HCV coinfected individuals in the country, we analyzed almost half of all newly HIV-1 diagnosed and notified cases between 2009 and 2019 for evidence of viremic and resolved HCV infections. The analyzed proportion mirrors very well the notified cases in their entirety regarding gender, mode of transmission and German or non-German origin.

Our sample material utilized in the study was residual serum or plasma from HIV-1 diagnoses. HIV-2 diagnoses are extremely rare in the country and were not analyzed [33]. The term coinfection is used here in a sense not restricted to simultaneous HIV/HCV acquisitions. The order of the acquisition could vary.

Since the applied screening ELISA detected both, HCV antibodies and HCV capsid-antigen, early HCV infections prior to antibody seroconversion are also detected. ELISA reactivity (HCV antigen, antibody or both) reflects therefore the antibody seroprevalence plus cases with early HCV infection. However, the number of ELISA-positive cases lacking antibody-reactivity can be assumed as low so that the proportion of positively tested cases is comparable to the antibody seroprevalence [24]. To refine the screening, additional assays were applied on 961/1,062 reactive samples and 44 (4.6%) ELISA-reactive cases subsequently tested negative by HCV-PCR and HCV-Western blot were excluded from further analysis. All obtained diagnostic results were examined on annual basis to analyze time trends.

The average prevalence of cases with an ongoing or a resolved HCV infection in our study was 6.2% (95% CI 5.4–7.0). This is about twenty times higher than the HCV seroprevalence of 0.3% reported for the general population examining samples collected between 2008 and 2011 in Germany [30, 34]. In the study by Poethko-Müller and colleagues, two thirds of the HCV reactive samples were found viremic which is similar to the average of 67% in our study. However, in 2009–2011 the average in our study was 71% and declined from 2012 onwards, reaching 52% in 2019 (see Fig. 3). It is probable that this decrease is caused by the introduction of the first generation of DAAs late in 2012 in Germany and other European countries and by the introduction of the second and much more potent DAA generation in 2014 [35]. This finding is in agreement with a similar increase we reported earlier from a cohort of HIV-1-positive patients in which the HCV incidence rate remained stable between 2009 and 2019 [36].

Although the rate of spontaneous resolution of an HCV infection is in general slightly higher in females [37], we did not see a significant difference between men and women in the proportion of active and resolved HCV cases and resolved infections. The reason might be a too low power because of fewer females in the study (3,235 cases (19.6%)) or a somewhat higher proportion of infections cleared by DAAs in males.

Known subpopulations of elevated risk for both HIV and HCV infections are MSM and foremost PWIDs [4, 38]. Remarkably, the percentage of active and resolved cases in MSM (2.6%) was significantly lower compared to HET (3.8%) and PWIDs (73.7%). We reported earlier a prevalence of 8.2% in MSM of the German HIV-1 seroconverter cohort and a comparable prevalence of 8.8% was found in an internet survey [39, 40]. Remarkably, most of the HCV-positive HIV-1 seroconverters acquired HCV after HIV-1 seroconversion [39]. Moreover, in a previous metanalysis an HCV prevalence of 0.5% (active and resolved) was published for HIV negative MSM and 8.8% for MSM with an HIV diagnosis in Germany [28]. The underlying reasons for this discrepancy are not well studied, but an association with sexual risk behavior can be assumed. Since the samples in our study were from MSM just diagnosed HIV-1-positive, an HCV prevalence of 2.6% is plausible. An HCV coinfection after HIV infection does not appear to be uncommon in MSM [28, 39, 40]. This is in line with data analyzing individuals with recently acquired HCV infections in the last decade in Germany [41–43]. Along these lines Graf and coworkers investigated patients with recent HCV infections and report that they occur primarily in PWID and in HIV coinfected MSM [41]. In MSM an association with sexual risk behavior was found [41].

Of interest is the prevalence of active and resolved HCV infections of 3.8% in HET in our study since heterosexual transmission of HCV is rarely documented [44]. This prevalence is higher than the reported seroprevalence in the general population in Germany (0.2%-1.9%, as mentioned above). As an underlying reason, we presume a higher risk behavior for HCV acquisition of individuals in the HET transmission group in our study compared to the average in the general population. This could reflect under-appreciated transmission risks within certain heterosexual networks for example those originating from HCV endemic regions. Furthermore, it cannot be ruled out that heterosexual transmission was reported due to stigma but another transmission route with a higher probability of HCV transmission was present.

In agreement with previous results is the share of active and resolved HCV cases of 73.7% in PWIDs [45–48]. In a meta-analysis, Sperle and coworkers reported earlier an HCV seroprevalence among PWIDs in Germany of 68.0% [32]. In PWIDs with HIV 82.1% are estimated to be HCV seropositive in the country, a situation similar to other Western-European countries [4]. Although PWIDs with HIV are the risk-group with the highest prevalence of active and resolved HCV cases, they had the lowest proportion of resolved HCV infection (29.5%) while MSM had the highest (40.0%). The difference between the pre- and post-DAA era is also the smallest for PWIDs (26.2% vs 30.5%). Altogether our findings indicate that PWIDs as subpopulation benefited the least from therapeutic options in the study period. To some extent, this might be a result of more frequent HCV-reinfections in this transmission group besides lower diagnostic and therapeutic care [49–51]. Overall, PWIDs in Germany and elsewhere suffer from structural or socio-economic barriers and other disadvantages such as stigmatization, limitations of low threshold services, frequent homelessness, imprisonment, mental health comorbidities as well as low HCV testing frequency, linkage to care deficits and low HCV status awareness [29, 52–56]. These circumstances are known to be responsible for preventing PWIDs from accessing and completing HCV therapy and mitigate reinfection rates. More studies and public health efforts are needed to resolve this.

About one third of the studied samples came from individuals with non-German origin. The prevalence of active and resolved HCV infections in those cases was twice as high as in cases from individuals of German origin and the proportion of resolved infections in those samples was significantly lower. Our data show that people from high HIV and HCV prevalence regions of Eastern Europe (especially from countries of the former Soviet Union) and Central Asia had an 8-fold higher proportion of active and resolved HCV-positive cases compared to samples from individuals of German origin and an approximately 4-fold higher proportion of PWIDs. These are regions with high HCV prevalence especially among PWIDs [4]. In contrast, individuals from Sub-Sahara Africa contributing 37.7% to the cases of non-German origin have a 4.3-fold lower prevalence of active and resolved HCV infections as the cases of German origin.

Our study is not without limitations. (i) The proportion of analyzed samples in the first two years of the study period and in 2014 is low and the prevalence determination therefore less robust. (ii) False positive ELISA results could only be identified in 961 of 1,062 (90.5%) ELISA-reactive samples due to lack of material for confirmatory testing of 101 cases. (iii) The 1:5 dilution associated with the elution of viruses and antibodies from the filter could hypothetically drive some samples below the detection level of the subsequent diagnostic, although this is in light of our previous studies regarded as unlikely [24].

Our study concludes with HIV cases from 2019. The subsequent COVID-19 pandemic led to a decrease in new HIV and HCV diagnoses in Germany in 2020–2021, followed by a catch-up effect with a sharp increase in 2022–2023 [57, 58]. For HCV, the inclusion of an HCV testing in the general health check-up at age 35 (check-up 35) in 2021 also contributed to this increase [57, 59]. More recently the impact of refugees from the Ukraine is noticeable [57]. To meet those challenges and to further reduce the proportion of active HCV cases among new HIV diagnoses and the prevalence of both infections in the long run, an intensified testing and treatment is mandatory, as well as a reinforcement of micro-elimination efforts in populations at high risk.

In conclusion, our data provide information on people with HCV coinfections at the time of HIV-1 diagnoses in Germany in a period of dramatic changes in HCV therapy options. It allows an early recognition of epidemiological trends, and can support the implementation of appropriately targeted prevention measures. These measures should focus on and prioritize PWIDs and individuals of non-German origin living with HIV by offering further options for low threshold counseling, testing and treatment in order to meet national and WHO elimination goals by 2030 [10, 19, 20].

## Electronic supplementary material

Below is the link to the electronic supplementary material.

Supplementary Material 1

## Data Availability

All datasets and materials are available from the corresponding author upon reasonable request.

## Abbreviations

CI: Confidence Interval
DAA: Direct-Acting Antivirals
DSS/DPS: Dried Serum or Plasma Spots
ELISA: Enzyme-linked Immunosorbent Assay
HCV: Hepatitis C Virus
HET: Heterosexual Mode of Transmission
HIV: Human Immunodeficiency Virus
IFN: Interferon
IgG: Immunoglobulin G
MSM: Men who have Sex with Men
PWID: People Who Inject Drugs
RKI: Robert Koch Institute
RT-qPCR: Quantitative Real-Time Polymerase Chain Reaction
