# Supplementary material for: Active and resolved HCV infections among people with new HIV-1 diagnoses in Germany, 2009–2019
Source: BMC Infect Dis. 2025 Nov 5;25:1511. doi: 10.1186/s12879-025-12025-8 (PMC12590741; doi:10.1186/s12879-025-12025-8)
Supplement: Supplementary file 1 — Supplementary Material 1 [file 12879_2025_12025_MOESM1_ESM.pdf]

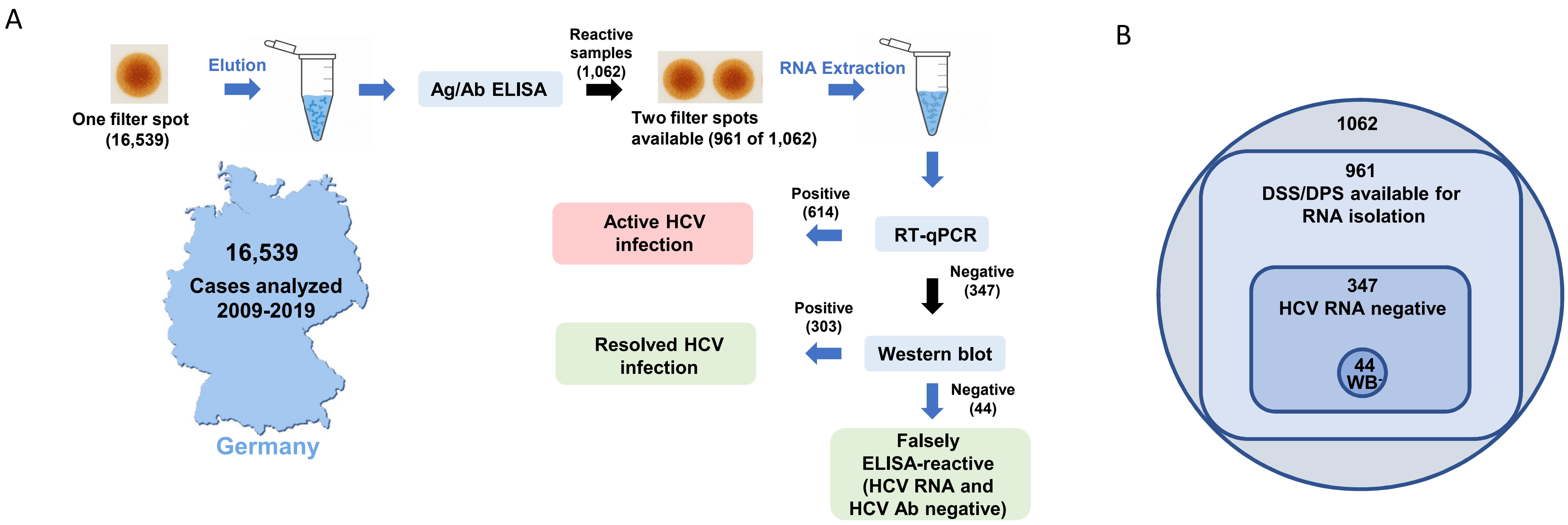

(A) **Schematic presentation of the study procedure and diagnostic analyzes in the laboratory**. Numbers in parentheses represent sample numbers. RNA-isolation has been performed from two additional filter spots. Such spots were available from 961 of 1,062 ELISA-reactive cases only. The Western blot has been performed with material eluted from the primary filter spot. Of the initially ELISA-reactive 1,062 cases, HCV antibodies or RNA could be confirmed in 917 cases. In 44 cases neither HCV antibodies nor HCV RNA was detected in the confirmation assays. A confirmation assay has not been performed for 101 cases due to a lack of material for RNA isolation.

(B) **Relevant HCV-related characteristics of the 1,062 initially ELISA-reactive cases**. The 44 RNA and Western blot negative (WB^-) samples were assumed as falsely ELISA-reactive due to the lack of confirmation by HCV-RT-qPCR and Western blot, leading to 1,018 HCV antigen- and/or antibody-reactive cases (active and resolved infections).

Additional file 1
